# Genetic variants in RIG-I-like receptor influences HCV clearance in Chinese Han population

**DOI:** 10.1017/S0950268819000827

**Published:** 2019-05-09

**Authors:** Xinyu Wu, Feng Zang, Mei Liu, Lingyun Zhuo, Jingjing Wu, Xueshan Xia, Yue Feng, Rongbin Yu, Peng Huang, Sheng Yang

**Affiliations:** 1 Department of Clinical Medicine, School of Basic Medical Sciences, Nanjing Medical University, Nanjing 211166, China; 2 Department of Epidemiology and Biostatistics, School of Public Health, Key Laboratory of Infectious Diseases, Nanjing Medical University, Nanjing 211166, China; 3 Department of Epidemiology, School of Public Health, Key Laboratory of Infectious Diseases, Nanjing Medical University, Nanjing 211166, China; 4 Department of Biostatistics, School of Public Health, Nanjing Medical University, Nanjing 211166, China; 5 Faculty of Life Science and Technology, Kunming University of Science and Technology, Kunming 650500, China

**Keywords:** Gene polymorphism, hepatitis C, RIG-I, spontaneous clearance

## Abstract

Human innate immune plays an essential role in the spontaneous clearance of acute infection and therapy of HCV. We investigated whether the SNPs in retinoic acid-inducible gene I-like receptor family were associated with HCV spontaneous clearance and response to treatment. To evaluate the clinical value of *DDX58* rs3824456, rs10813831 and rs10738889 genotypes on HCV spontaneous clearance and treatment response in Chinese Han population, we genotyped 1001 HCV persistent infectors, 599 participants with HCV natural clearance and 354 patients with PEGylated interferon-*α* and ribavirin (PEG IFN-*α*/RBV) treatment. People carrying rs10813831-G allele genotype were more liable to achieve spontaneous clearance than the carriage of the T allele (dominant model: adjusted OR 1.35, 95% CI 1.08–1.71, *P* = 0.008). In rs10738889, the rate of persistent infection was significantly lower in patients with the TC genotype compared to those with TT genotype (dominant model: adjusted OR 1.36, 95% CI 1.06–1.74, *P* = 0.015). Multivariate stepwise analysis indicated that rs10738889, age, alanine aminotransferase (ALT) and aspartate aminotransferase (AST) were independent predictors for HCV spontaneous clearance. However, there were no significant differences in the three selection SNPs between the non-SVR group and the SVR group. These results suggest the *DDX58* rs10813831 and rs10738889 are associated with spontaneous clearance of HCV, which may be identified as a predictive marker in the Chinese Han population of HCV.

## Introduction

Hepatitis C is a worldwide contagious disease caused by the hepatitis C virus (HCV). Globally, it is estimated that a total of 71 million people have chronic hepatitis C infection according to the World Health Organization. HCV is spread primarily by blood-to-blood contact including intravenous drug use; transfusions, poorly sterilised medical equipment and needle stick injuries in healthcare. It may also be transmitted from an infected mother to her baby during labour. Early on chronic infection, no typical symptoms are shown. However, it often leads to cirrhosis, even hepatocellular carcinoma after many years [1].

Though direct acting antiviral agents, like sofosbuvir, daclatasvir and the sofosbuvir/ledipasvir combination, were already approved in western countries, the combination therapy of pegIFN-*α*/RBV is still the most effective treatment in developing countries such as China due to economic issues and curative concerns [2, 3]. Environment, viral and host factors have been proved to be the influential factor in HCV spontaneous clearance and treatment response [4]. The variation of host immune response against HCV may affect the results of HCV infections the most [5, 6].

Human innate immune plays an important role in inhibiting HCV replication and diffusion [7]. PRRs (pattern recognition receptors) function as a pattern recognition receptor that identifies virus RNA through PAMPs (pathogen-associated molecular patterns) [8, 9]. RIG-I (retinoic acid-inducible gene I) is a part of the RIG-I-like receptor (RLR) family, which also includes MDA5 and LGP2 [2, 10]. RIG-I and MDA5 each contain two N-terminal caspase activation and recruitment domains (CARD), which will initiate the anti-viral signalling pathway [4, 11]. LGP2 does not have CARD-domains and therefore does not positively induce signalling pathway nor participate in viral detection [12]. Instead, LGP2 modulates the other two RLRs through negative inhibition [13]. It is known that LGP2 binds a repressor domain on the RIG-I C-terminal region to suppress RIG-1 signalling and down-regulate the viral response [14, 15].

We speculate that single nucleotide polymorphisms (SNPs) in RLR family can lead to different levels of immune response and result in the individual outcomes of HCV infection. It is known that RIG-I is an RLR dsRNA helicase enzyme that is encoded (in humans) by the *DDX58* gene. Thus, through researching the relationship between *DDX58* gene polymorphism and HCV immunity level, we could discover how RIG-I affects HCV infection.

Our study is intended to investigate whether several variants of *DDX58* gene are associated with the outcomes of HCV infection and response to treatment. Thus, we generated data from a high-risk population to systematically examine the relationship between three SNPs (*DDX58* rs3824456, rs10813831 and rs10738889) and HCV clearance.

## Methods

### Patients

A total of 1600 participants, who had not previously received chemotherapy, were enrolled in this research This population included 324 cases of drug users in the Nanjing compulsory detoxification centre from May to December 2006, 187 haemodialysis patients from nine haemodialysis centres in southern China during October 2008 and January 2010, and 1089 former paid-blood donors were gathered in Zhenjiang of Jiangsu province from April 2011 to April 2015. Those who spontaneously clear the HCV without treatment were defined as the spontaneous clearance group. Oppositely, a persistent state in which the virus is present in the host without being cleared fell into persistent infection groups.

Three hundred and fifty-four treatment-naïve CHC patients were recruited from Jurong People's Hospital (Jurong, China) from January 2011 to October 2013, aiming to evaluate the factors that impacted on response to anti-viral therapy. Eligibility criteria for therapy included (1) age over 18, (2) detectable HCV RNA in serum over a span of more than 6 months of treatment initiation, (3) negative in hepatitis B surface antigen and (4) clear in other causes of liver disease.

Each participant was interviewed face-to-face by using a structured questionnaire to collect information on demographic data and environmental exposure history.

### Viral test

After the interview, every individual donated 5 ml of venous blood samples for serological tests and DNA extraction. The serum and peripheral blood mononuclear cells were separated and stored at −70 °C until assay. Sera and HCV antibody were detected by ELISA (Beijing Wantai Biological Pharmacy Enterprise Co., Ltd., Beijing, China) and extract DNA from peripheral blood by phenol chloroform extraction. HCV RNA was removed from the serum by using PT-PCR Kit (TaKaRa biotechnology Co., Ltd., Dalian, China). The authors followed up these participants and monitored the viral load at weeks 0, 4, 12, 24 and 48 during treatment, and post-treatment week 24.

### SNPs selection and genotypic assays

According to our previous work, information regarding SNPs in candidate genes (*DDX58*) was acquired from the NCBI dbSNP database (http://www.ncbi.nlm.nih.gov/SNP) and the Chinese Han population database of HapMap (hrrp://hapmap.org). All the SNPs were filtered with the following criteria: (1) minimum allele frequency ⩾0.05 in Chinese Han population; (2) Hardy–Weinberg equilibrium (HWE) test *P* value ⩾ 0.05. After the above steps, three SNPs (*DDX58* rs3824456, rs10813831 and rs10738889) were chosen for genotyping. DNA extraction was done by protease K digestion and phenol–chloroform purification. Ultraviolet spectrophotometry was used to identify the DNA concentration. Genotype polymorphism was genotyped by the TaqMan allelic discrimination assay with the ABI PRISM 7900HTsequence detection system (Applied Biosystems, San Diego, CA, USA). For quality control, each 384-well format was assigned to two blank controls and five replicate samples. (PCR was performed at 50 °C for 2 min, 95 °C for 10 min, 95 °C for 15 s and 60 °C for 1 min then repeated for 40 cycles.)

### Statistical analysis

All data analyses were operated with Stata/SE (V.12.0 for Windows). Demographic characteristics of individuals in each group were calculated by the Student's *t* test or the *χ*^2^ test with a two-tailed *P* value. Co-dominant, dominant and additive genetic models were used in the analysis of each SNP, dominant model stands for (homozygous type + hybrid type) *vs.* wild type; recessive model stands for homozygous type *vs.* (hybrid type + wild type) and additive model stands for hybrid type *vs.* homozygous type *vs.* wild type.

Multivariate logistic regression was used to analyse the association between genetic with HCV spontaneous clearance and clinical data by calculating the OR and 95% CI adjusted for age, gender, ALT, AST in first population and age, gender, HCV RNA, albumin (ALB), platelets, *α* foetal protein (AFP) in clinical population. A forward elimination stepwise regression analysis containing all variables was used to determine the prediction factors for spontaneous clearance. A two-tailed test with a *P* value < 0.05 was regarded as statistically significant in all analyses.

## Result

### Basic characteristics of the population with different infection outcomes

Baseline characteristics in the subject with different HCV infection outcomes are shown in Table 1. According to the detection result of anti-HCV and HCV RNA content, our survey respondents were divided into two sets: 599 subjects with spontaneous clearance and 1001 persistent HCV infectious. The two groups were incomparable in terms of gender (*P* = 0.293). However, statistically significant differences were observed in AST, ALT and age (all *P* < 0.001). These data show that the patients who are over 50 years old have less chance to achieve spontaneous clearance, and the one with spontaneous clearance has a higher level of AST and ALT than those with persistent infection.

**Table 1.** Demographic characteristics in subjects with different HCV infection outcomes

Variables	Spontaneous clearance	Persistent infection	*P* value
	*n* = 599	*n* = 1001	
Age (year)	49.66 ± 13.60	54.10 ± 11.84	<0.001
<50	255 (42.57)	280 (27.97)	<0.001
⩾50	344 (57.43)	721 (72.03)	
Gender (%)			0.293
Male	217 (36.29)	336 (33.70)	
Female	381 (63.71)	661 (66.30)	
AST (%)	33.72 ± 32.73	47.31 ± 37.79	<0.001
<40	474 (79.13)	567 (56.64)	<0.001
⩾40	125 (20.87)	434 (43.36)	
ALT (%)	35.43 ± 44.64	46.51 ± 46.30	<0.001
<40	469 (78.30)	606 (60.54)	<0.001
⩾40	130 (21.70)	395 (39.46)	

AST, aspartate transaminase; ALT, alanine aminotransferase.

### Associations of selected gene polymorphisms with HCV spontaneous clearance

The genotype distribution of the three SNPs between HCV patients with persistent infection and subjects with spontaneous clearance is in Table 2. The observed genotype frequencies for the SNPs in subjects with spontaneous HCV clearance were all in HWE (*P* > 0.05). Co-dominant, dominant, recessive and additive genetic models were utilised in the analysis of each SNP after adjustment with age, gender, ALT and AST. Logistic regression analyses showed that the distribution of *DDX58* rs10813831 and rs10738889 was associated with spontaneous clearance. Subjects with an rs10813831-GG genotype seem to be more likely to achieve spontaneous clearance than those with hybrid GT genotype (co-dominant model: adjusted OR 1.42, 95% CI 1.11–1.82, *P* = 0.005). Compared with the reference carrying rs10813831-G allele genotype, the carriage of the T allele had a significantly increased likelihood of continuing a persistent infection (dominant model: adjusted OR 1.35, 95% CI 1.08–1.71, *P* = 0.008; additive model: adjusted OR 1.29, 95% CI 1.07–1.56, *P* = 0.007). In rs10738889 SNPs, the rate of persistent infection was significantly lower in patients with the CC genotype compared to those with TT and TC genotypes (dominant model: adjusted OR 1.35, 95% CI 1.08–1.71, *P* = 0.008; dominant model: adjusted OR 1.36, 95% CI 1.06–1.74, *P* = 0.015). The co-dominant model analyses indicated that the wild genotype (rs10738889-TT) has a high probability to achieve spontaneous clearance than the heterozygote (rs10738889-TC) (adjusted OR 1.41, 95% CI 1.09–1.82, *P* = 0.008). Nevertheless, other SNPs (rs3824456) have no statistical correlation, suggesting an effect on hepatitis C spontaneous clearance (all *P* > 0.05).

**Table 2.** Distribution of *DDX58* genotypes among subjects with different HCV infection outcomes

Genotype	Spontaneous clearance	Persistent infection	OR (95% CI)	*P* value
rs3824456				
GG	295 (51.66)	501 (53.30)	1.00	–
GA	232 (40.63)	362 (38.51)	0.97 (0.77–1.22)	0.784
AA	44 (7.71)	77 (8.19)	1.21 (0.74–1.69)	0.584
Dominant			0.96 (0.78–1.19)	0.740
Recessive			1.12 (0.75–1.66)	0.592
Additive			1.02 (0.86–1.21)	0.824
rs10813831				
GG	409 (69.91)	553 (56.89)	1.00	–
GT	145 (24.79)	350 (36.01)	1.42 (1.11–1.82)	0.005
TT	31 (5.30)	69 (7.10)	1.41 (0.89–2.24)	0.138
Dominant			1.41 (1.12–1.77)	0.004
Recessive			1.25 (0.79–1.95)	0.339
Additive			1.29 (1.07–1.56)	0.007
rs10738889				
TT	200 (34.97)	288 (29.60)	1.00	–
TC	239 (41.78)	477 (49.02)	1.36 (1.06–1.74)	0.015
CC	133 (23.25)	208 (21.38)	1.16 (0.86–1.56)	0.319
Dominant			1.31 (1.06–1.64)	0.014
Recessive			0.99 (0.76–1.28)	0.934
Additive			1.10 (0.95–1.28)	0.204

Logistic regression analyses adjusted for age, gender, ALT and AST.

### Prediction of independent factor affecting the prognosis of HCV infection

To further explore the independent influencing factors that affect the HCV infection, statistically significant baseline variables (age, gender, ALT, AST), the rs10738889 and rs10813831 SNPs were brought into the stepwise regression analysis; the result suggested that rs10738889, age, ALT and AST were independent predictors of the disease (all *P* < 0.05). The concrete result was shown in Table 3.

**Table 3.** Multivariate stepwise regression analysis for independent factors of HCV chronicity

Variables	Coef.	s.e.	OR (95% CI)	*P* value
rs10738889	0.16	0.05	1.17 (1.05–1.29)	0.002
ALT	1.36	0.14	3.89 (2.98–5.10)	<0.001
Age	0.40	0.10	0.67 (0.55–0.82)	<0.001
AST	1.25	0.13	3.49 (2.71–4.50)	<0.001

AST, aspartate transaminase; ALT, alanine aminotransferase.

### Stratified analysis among subjects with different HCV infection outcomes

According to the independent predictors of HCV infection, a stratified analysis was performed in Table 4. The results for *DDX58* rs10738889 indicated, when compared with the TT genotype, a significantly increased risk of HCV chronicity in C alleles in the subgroup among patients with AST level higher than 40 (adjusted OR 1.68, 95% CI 1.13–2.50; *P* = 0.010) and the subgroup whose ALT level over 40 (adjusted OR 1.49, 95% CI 1.11–2.01; *P* = 0.007). However, no significant effect of rs10738889 on HCV spontaneous clearance was found in different age groups. The heterogeneity test in all three subgroups proved that the level of ALT, age, level of AST are confounding factors in the association of HCV infection.

**Table 4.** Stratified analysis of rs10738889 genotypes among subjects with different HCV infection outcomes

Stratified characteristics	Spontaneous clearance			Persistent infection			OR (95% CI)	*P*^a^	*P*^b^
	TT	TC	CC	TT	TC	CC			
Age									0.045
<50	59 (40.69)	41 (28.28)	45 (31.03)	35 (28.23)	48 (38.71)	41 (33.06)	1.26 (0.94–1.71)	0.126	
⩾50	141 (33.02)	198 (46.37)	88 (20.61)	253 (29.80)	429 (50.53)	167 (19.67)	1.05 (0.89–1.25)	0.546	
ALT									0.009
<40	140 (31.25)	197 (43.97)	111 (24.78)	169 (28.79)	297 (50.60)	121 (20.61)	1.19 (0.96–1.47)	0.109	
⩾40	60 (48.39)	42 (33.87)	22 (17.74)	119 (30.83)	180 (46.63)	87 (22.54)	1.68 (1.13–2.50)	0.010	
AST									0.0265
<40	148 (32.67)	195 (43.05)	10 (24.28)	169 (30.90)	265 (48.45)	113 (20.66)	1.23 (0.99–1.52)	0.065	
⩾40	52 (43.70)	44 (36.97)	23 (19.33)	119 (27.93)	212 (49.77)	95 (22.30)	1.49 (1.11–2.01)	0.007	

AST, aspartate transaminase; ALT, alanine aminotransferase; OR, odds ratios; 95%CI, 95% confidence intervals.

a The *P*-value was calculated by the logistic regression model, adjusted by age, ALT and AST in addictive model (GG *vs*. GT *vs*. TT for rs10738889).

b The *P*-value was the result of the heterogeneity test.

### Baseline clinical characteristics of CHC patients after pegIFN-*α*/RBV therapy

For further exploration of the deeper relationship between the selected SNPs and HCV, we explored the influence of selected SNPs (rs3824456, rs10813831 and rs10738889) on the response to pegIFN-*α*/RBV (interferon-*α* combined with ribavirin) therapy in CHC patients. On the basis of the HCV RNA level, we divided these patients into two groups after the 48 weeks' treatment, which included 120 cases who did not achieve an SVR (non-SVR group) and 234 cases achieving SVR (SVR group). The SVR rate was 66.10%. The clinical characteristics were compared between the two groups in Table 4. Statistically significant differences were observed in the level of baseline RNA, ALB, GLU (glucose), AFP, platelets (all *P* < 0.05). These indicators could be the influence factors of the CHC patients who achieved SVR. However, other characteristics showed no significant difference between the two groups (*P* > 0.05).

### Effect of polymorphisms of selected genes on treatment response

Four genetic regression models (co-dominant additive, dominant and recessive models) were applied to detect the possible association of rs3824456, rs10813831 and rs10738889 with treatment response in CHC patients. The genotype distribution of the three SNPs among CHC patients is summarised in Table 5. The logistic regression analysis, adjusted by ALB, GLU, AFP, platelets and baseline RNA, showed no significant differences for all the three selected genes between the non-SVR group and the SVR group (all *P* > 0.05).

**Table 5.** Baseline clinical characteristics of chronic hepatitis C patients

	N-SVR	SVR	
Variables	*n* = 120
		*n* = 234	*P* value
Age (year)	53.54 ± 8.09	53.70 ± 8.58	0.870
<50	36 (30.00)	74 (31.62)	0.755
⩾50	84 (70.00)	160 (68.38)	
Sex (%)			0.697
Male	29 (24.17)	61 (26.07)	
Female	91 (75.83)	173 (73.93)	
AST ⩾ 40 U/L (%)	69 (57.50)	124 (52.99)	0.420
ALT ⩾ 40 U/L (%)	78 (65.00)	140 (59.83)	0.344
Baseline RNA	6.15 ± 0.81	5.95 ± 0.90	0.046
TP(g/L)	77.76 ± 5.90	78.28 ± 5.97	0.437
ALB(g/L)	42.66 ± 4.22	43.71 ± 4.07	0.024
GLU (mmol/L)	6.34 ± 1.64	5.76 ± 1.54	0.001
AFP (ng/mL)	13.12 ± 30.00	6.23 ± 15.15	0.007
TSH (nmol/L)	2.80 ± 1.78	3.84 ± 7.68	0.145
HGB (g/L)	132.92 ± 17.50	134.31 ± 16.15	0.456
Platelets (10^9^/L)	121.72 ± 59.85	135.94 ± 52.75	0.023
WBC (10^9^/L)	4.87 ± 2.63	5.00 ± 1.76	0.578

N-SVR, non-sustained virological response; SVR, sustained virological response; AST, aspartate transaminase; ALT, alanine aminotransferase; TP, total protein; ALB, albumin; GLU, glucose; AFP, *α* foetal protein; TSH, thyrotropin; HGB, haemoglobin; WBC, white blood cell.

## Discussion

Hepatitis C has always been regarded as the ‘silent epidemic’. Approximately 399 000 people die from hepatitis C every year, mostly from cirrhosis and hepatocellular carcinoma (WHO). HCV causes both acute and chronic infection. Acute HCV infection is usually asymptomatic, and the risk of chronicity is so high that the remaining 60–80% of people will develop chronic HCV infection, but there is still no enough evidence to explain this mechanism. About 15–45% of infected individuals spontaneously clear HCV within 6 months without any treatment, the difference between individuals related to spontaneous clearance and persistent infection is worth discussing.

Due to the joint effects of viral, individually human genotype and high-risk behavioural factors, HCV-infected patients often exhibit distinct clinical outcomes. According to some reports, human immune-related genes play an important part in the HCV clearance; many studies on human genetic polymorphism and clearance of HCV have been carried out at home and abroad [16, 17]. Thus, it is a valuable discussion between the HCV clearance and gene polymorphism.

The association between gene polymorphisms in the RLR family and HCV infection has been little researched. The RLRs had been proved that it had viral ligand specificity for paramyxo viruses, vesicular stomatitis virus and influenza virus [14, 18]. The studies assessing the association between the RLR family and HCV spontaneous clearance are still in the preliminary stages.

It is for the first time to take the Chinese Han population as samples to demonstrate the relationship between variants in RLRs and HCV treatment response. In the previous study, we learned that the infection of HCV was affected by host immune, and the RLRs played a major role in non-specific immunity [12]. Our target gene *DDX58*, featuring the conservative motif Asp-Glu-Ala-Asp(DEAD), is a recognised RNA helicase involved in a number of cellular processes including RNA binding and alteration of RNA secondary structure [19]. RIG-I is associated with the viral double-stranded (ds) RNA recognition and the regulation of immune response. *DDX58* rs3824456, rs10813831 and rs10738889 are all located in the intron region of the gene according to the NCBI database. The associations between these SNPs and HCV infection were investigated in co-dominant, dominant and additive models.

The result showed that *DDX58* rs10813831 and rs10738889 were associated with HCV spontaneous clearance. Additionally, stepwise regression model showed that rs10738889, sex, age and AST were independent predictors of hepatitis C pathogenesis. Further stratification analysis showed that the TT genotype in the subgroups, with AST or ALT level higher than 40, had an increased risk to achieve spontaneous clearance than those carrying with C alleles. Compared with the patients with ALT and AST level lower than 40, which had no statistical differences, the higher groups indicated that rs10738889 may play a bigger role in acute liver cell injury. Moreover, our study found that subjects with the heterozygous rs10738889-CT genotype were more prone to HCV infection. It is reported that this SNP is associated with measles-specific antibody variations, which may play the same role in hepatitis C infection [20]. Coincidentally, the other SNPs with statistical signification also influence the measles immunity [21].

Different from other selected SNPs, the missense mutation in the rs10813831 (G→T) has a strong possibility of affecting its function. Our result showed that the patients who carry rs10813831-G allele genotype had a significantly decreased probability of continuing a persistent infection than the carriers of the T allele. According to the previous study, *DDX58* rs10813831 non-GG (*vs.* GG) was associated with more severe recurrent HCV, which is accorded with our conclusion [22]. Moreover, there is an article that revealed that rs3824456 and rs10813831 both associate with Rubella vaccine-induced cellular immunity [23]. However, results were different from the two gene locus in our study. This could be because rs3824456 has an impact on the variations in TNF-*α* secretion levels and rs10813831 is associated with the variations in IL-6 secretion levels [23].

Our study also has some potential limitations. First, the biological mechanism by which RLRs affect the treatment response has not yet been well established. This study targeted only three SNPs as the HCV outcomes influence the factor. Although the rs10738889 was the independent factor of HCV infection outcomes, the discrepancy from the treatment phase still needed further exploration to discover which SNPs really make the differences. Second, we ignored the effects of genotype. It may slightly affect the accuracy of our study. It is worth noting that Chinese HCV infectors are most infected with genotype 1b hepatitis C [24]. However, further studies are still required to add genotypes into adjustment as confounding factors to get a more reliable result. Third, we did not include the IL28B variation in the statistical calculation as an immunologic parameter due to the lack of complete data. What is more, the direct acting antiviral combination is getting more effective response treatment internationally. However, owing to various restrictions, the pegIFN-*α*/RBV therapy was used as the first-line treatment in China. It might be an important factor that leads to the indistinctive consequence.

**Table 6.** Distribution of *DDX58* genotypes among chronic hepatitis C patients

Genotype	N-SVR	SVR	SVR rate (%)	OR (95% CI)	*P* value
rs3824456					
	65 (55.08)	129 (56.83)	66.49	1.00	–
	46 (38.98)	82 (36.12)	64.06	0.91 (0.55–1.52)	0.724
	7 (5.94)	16 (7.05)	69.57	1.01 (0.32–3.14)	0.991
Dominant				0.88 (0.54–1.44)	0.623
Recessive				1.04 (0.34–3.19)	0.939
Additive				0.95 (0.63–1.44)	0.812
rs10813831					
	17 (89.17)	209 (91.27)	92.48	1.00	–
	6 (5.00)	9 (3.93)	60.00	0.75 (0.23–2.41)	0.624
	7 (5.83)	11 (4.80)	61.11	0.77 (0.27–2.18)	0.623
Dominant				0.75 (0.34–1.67)	0.479
Recessive				0.77 (0.27–2.18)	0.625
Additive				0.86 (0.53–1.39)	0.533
rs10738889					
	50 (43.10)	99 (44.00)	66.44	1.00	–
	55 (47.41)	97 (43.11)	63.82	0.88 (0.52–1.47)	0.615
	11 (9.49)	29 (12.89)	72.50	1.50 (0.62–3.59)	0.367
Dominant				0.97 (0.60–1.58)	0.910
Recessive				1.63 (0.71–3.75)	0.247
Additive				1.08 (0.75–1.57)	0.670

SVR, sustained virological response; N-SVR, non-sustained virological response.

Logistic regression analyses are adjusted for ALB, GLU, AFP, platelets, baseline RNA.

Correspondingly, some advantages of our study should not be ignored. This study first validated the association between *DDX58* and HCV response among such big sample capacity in China. This treatment cohort is credible since all patients were only infected with HCV, without co-infection with HBV/HIV and other causes of liver disease, and were enrolled from the same area at the same time, so it can better explore the natural history of HCV. Additionally, three kinds of high-risk population are inserted into our study to make our research all-sided. We believe that with the discovery of more and more genes related to the outcomes and treatment outcomes of HCV infection, the clearance and cure of HCV will be imminent.

In conclusion, *DDX58* rs10738889-C allele and rs10813831-G were associated with a decreased risk of HCV spontaneous clearance. The RLR family do play a role in HCV clearance in the Chinese Han population.

## Supplementary material

For supplementary material accompanying this paper visit https://doi.org/10.1017/S0950268819000827. click here to view supplementary material
